# Heart rate variability and DNA methylation levels are altered after short-term metal fume exposure among occupational welders: a repeated-measures panel study

**DOI:** 10.1186/1471-2458-14-1279

**Published:** 2014-12-16

**Authors:** Tianteng Fan, Shona C Fang, Jennifer M Cavallari, Ian J Barnett, Zhaoxi Wang, Li Su, Hyang-Min Byun, Xihong Lin, Andrea A Baccarelli, David C Christiani

**Affiliations:** Department of Environmental Health, Harvard School of Public Health, Boston, MA 02115 USA; New England Research Institute, Watertown, MA USA; University of Connecticut Health Center, Farmington, CT USA; Department of Biostatistics, Harvard School of Public Health, Boston, MA USA; Department of Epidemiology, Harvard School of Public Health, Boston, MA USA; Pulmonary and Critical Care Unit, Massachusetts General Hospital/Harvard Medical School, Boston, MA USA

## Abstract

**Background:**

In occupational settings, boilermakers are exposed to high levels of metallic fine particulate matter (PM_2.5_) generated during the welding process. The effect of welding PM_2.5_ on heart rate variability (HRV) has been described, but the relationship between PM_2.5,_ DNA methylation, and HRV is not known.

**Methods:**

In this repeated-measures panel study, we recorded resting HRV and measured DNA methylation levels in transposable elements Alu and long interspersed nuclear element-1 (LINE-1) in peripheral blood leukocytes under ambient conditions (pre-shift) and right after a welding task (post-shift) among 66 welders. We also monitored personal PM_2.5_ level in the ambient environment and during the welding procedure.

**Results:**

The concentration of welding PM_2.5_ was significantly higher than background levels in the union hall (0.43 mg/m^3^ vs. 0.11 mg/m^3^, p < 0.0001). The natural log of transformed power in the high frequency range (ln HF) had a significantly negative association with PM_2.5_ exposure (β = -0.76, p = 0.035). pNN10 and pNN20 also had a negative association with PM_2.5_ exposure (β = -0.16%, p = 0.006 and β = -0.13%, p = 0.030, respectively). PM_2.5_ was positively associated with LINE-1 methylation [β = 0.79%, 5-methylcytosince (%mC), p = 0.013]; adjusted for covariates. LINE-1 methylation did not show an independent association with HRV.

**Conclusions:**

Acute decline of HRV was observed following exposure to welding PM_2.5_ and evidence for an epigenetic response of transposable elements to short-term exposure to high-level metal-rich particulates was reported.

**Electronic supplementary material:**

The online version of this article (doi:10.1186/1471-2458-14-1279) contains supplementary material, which is available to authorized users.

## Background

Numerous air pollution studies have shown that both acute and cumulative exposures to fine particulate matter (PM_2.5_) are associated with increased risk of adverse cardiovascular events, such as the onset of atrial fibrillation (AF), incidence and recurrences of myocardial infarction, heart failure and stroke, and mortality from cardiovascular disease [1–4]. Heart rate variability (HRV) has been used as an early disease marker of adverse cardiovascular outcomes and as an indicator of cardiac autonomic function. It is believed that reductions in HRV alter the heart’s ability to properly respond to external signals, leading to myocardial infarction [5].

In occupational settings, welders are exposed to high levels of PM_2.5_ generated during the welding process. The metal components of PM_2.5_ play an important role in its toxicity [6]; metal-rich particle exposure related to welding has been associated with increased systemic inflammation, inflammation-related endothelial dysfunction, and elevated oxidative stress [7, 8]. For example, C-reactive protein (CRP), an inflammatory risk factor of endothelial dysfunction, is associated with fatal and non-fatal coronary artery disease (CAD) events in a healthy population [9, 10]. Studies have shown an association between increased circulating levels of CRP and decreased HRV in middle-aged men free of CAD, which suggests a relationship between ANS dysfunction induced and systemic inflammation [11]. Previous studies from the same cohort have reported an association between HRV and welding PM_2.5_ levels [12] and the concentration of various metal components of welding fumes [13].

Not only is inflammation associated with PM_2.5_, epigenetic changes have been implicated in PM-related conditions [14]. Epigenetic patterns are known to be sensitive to environmental exposures throughout the lifespan [15]. In a previous study, acute and chronic exposure to welding PM_2.5_ was associated with altered gene methylation [16]. DNA methylation in transposable elements, such as Alu and long interspersed nuclear elements-1 (LINE-1), which has been shown to be altered by PM_2.5_ exposure [17, 18].

Alu and LINE-1 elements are widely represented across the human genome and DNA methylation in which maintains transcriptional inactivation and integrity of the genome [19]. Studies show that decreased methylation in LINE-1 elements is associated with ischemic heart disease and stroke, as well as cardiovascular disease risk factors, such as high levels of low-density lipoprotein (LDL) and low levels of high-density lipoprotein (HDL) [20–23]. Decreased methylation has also been shown to be associated with increased vascular cell adhesion molecule-1 (VCAM-1), a biomarker of vascular inflammation [20–23], which suggests a potential functionality of DNA methylation in cardiovascular disease. A study has further reported that genetic variations in the methionine cycle affect heart rate variability which suggests the role of methylation in cardiac autonomic dysfunction and lower intake of nutritional methyl supplement is associated with the negative effect of PM_2.5_ on HRV [24]. However, whether DNA methylation levels in systemic circulation mediate the PM-induced cardiovascular effect measured in HRV is not known.

To investigate the cardiac and epigenetic changes in response to metallic welding fume exposure, we investigated the short-term effect of welding PM_2.5_ on resting HRV and blood DNA methylation in Alu and LINE-1 elements in a cohort of 66 male occupational welders. In addition, we tested the association between PM_2.5_-induced DNA methylation changes and HRV to characterize the mediation effect of DNA methylation on the relationship between welding PM_2.5_ and heart rate variability.

## Methods

### Study subjects

We recruited 66 male boilermakers free of cardiovascular disease at the Boilermaker Union Local 29, located in Quincy, Massachusetts, in a repeated-measures panel study. Written informed consent to participate in the study was obtained from each subject. The study protocol was approved by the Institutional Review Board (IRB) of the Harvard School of Public Health and the summary of population characteristics was shown in Table 1. In the union hall, there is a large, temperature-controlled classroom used by workers for break and it is outfitted with ten workstations in a separate area where the welders perform welding, cutting, and grinding tasks. Boilermakers primarily perform shielded metal arc and gas metal arc welding using base metals of mild steel (manganese alloys) and stainless steel (manganese, chromium, and nickel alloys) with electrodes composed mainly of iron with 1-5% proportions of manganese.

**Table 1:** **Summary of population characteristics**

Characteristics (n = 66)		Mean (SD)
Age (years)		41 (12.2)
Range		21-71
BMI (kg/m^2^)^a^		28.4 (5.0)
Range		16.9-38.7
Race	N	%
White	55	83
Black	6	9
Hispanic	3	5
Asian	2	3
Male	66	100
Current smoker ^a^	28	43.1

^a^ n = 65 due to missing questionnaire information.

### Study design

Six sampling cycles were performed from January 2010 to June 2012. Participants were recruited on multiple sampling cycles to perform welding. However, it was not a balanced design study and subjects had randomly missing sampling cycles. At each sampling cycle, personal air particulate exposure was monitored under work environment with and without welding activities to compare the effect of particle exposure generated during an average of five hours of welding to the background environment in the classroom with no direct welding fume exposure. Blood samples and resting HRV recordings were obtained before and after welding. A work log was used to collect personal information on work shift length, workstation, and exposure to secondhand smoke. A modified American Thoracic Society respiratory and cardiac questionnaire was used to collect medical history and medication use, demographics, and lifestyle information including smoking status and occupational history [25, 26].

### Particle exposure assessment

Real-time personal particle exposure was continuously monitored using the light-scattering technology of a DustTrak™ Aerosol Monitor (TSI, Inc., St. Paul, MN) fitted with an inlet impactor designed to separate particles with a median aerodynamic diameter of less than 2.5 μm (PM_2.5_). Each monitor received daily flow and calibration checks as well as yearly calibration performed by the manufacturer. Gravimetric analysis was not applied because a previous study of the same occupational cohort indicated good agreement between DustTrak™ and gravimetric measurements [27, 28]. Each monitor was placed in a padded pouch with the inlet tubing secured to the participant’s shoulder around the nasal breathing area. The DustTrak™ records PM_2.5_ concentration readings every ten seconds and calculates 1-min averages throughout the course of the welding shift. The DustTrak™ was worn during the welding shift to quantify PM_2.5_ exposure generated by welding, as well as over a similar period of time during the previous day, while the personal background environmental exposure was monitored in the absence of welding.

### Heart rate variability measurement

Continuous heart activity was recorded using a standard three-channel, seven-lead electrode Holter monitor (GE™ SEER Light Compact Digital Holter Recorder). Research staff helped participants to prepare for monitoring by shaving hair from each electrode site on the chest if necessary, and cleaning each electrode site with alcohol to improve conductivity. Heart rate variability was analyzed in the time and frequency domains. Time domain analysis calculated several measures of normal-to-normal (NN) intervals, including the standard deviation of the NN intervals (SDNN), square root of the mean squared difference of successive NN intervals (rMSSD), and the proportion of the number of interval differences of successive NN intervals greater than 10 and 20 milliseconds over the total number of NN intervals (pNN10 and pNN20). Frequency domain analysis evaluated how the power (variance) was distributed as a function of frequency and its fluctuation. Resting HRV was analyzed for power spectral density in the low frequency (0.04–0.15 Hz, LF), and power in the high frequency (0.15–0.40 Hz, HF) [29]. SDNN, rMSSD, pNN10, pNN20, LF, and HF was calculated over a five minute resting period during which participants were seated both before and after welding work. With the exception of pNN10 and pNN20, all other measurements were not normally distributed, and thus were natural log (ln)-transformed before data analysis.

In order to control the circadian variation of HRV, participants were encouraged to participate the baseline examination on non-welding days prior to welding days. A subgroup (n = 49) of the participants were monitored on non-welding days.

### DNA methylation analysis

Blood samples were collected from each participant before and right after welding work. Whole blood samples were collected by venous phlebotomy in EDTA tubes and promptly centrifuged on site for 15 min. After plasma was transferred, blood buffy coats were stored on dry ice for DNA extraction using a Gentra Autopure LS Large Sample Nucleic Acid Purification System (QIAGEN Company, Venlo, Limburg, Netherlands).

DNA methylation analyses of Alu and LINE-1 elements were performed on bisulfite-treated DNA by quantitative PCR pyrosequencing using the EZ DNA Methylation Gold Kit (Zymo Research, Orange, CA, USA) as previously described in detail [30] (See Additional file 1: Table S1). For all assays, we used built-in controls to verify bisulfite conversion efficiency. We tested the proportion of 5 methyl-cytosine (5mC) at each of five CpG dinucleotide positions of Alu and four CpG positions of LINE-1 and calculated the overall mean level of methylation for both Alu and LINE-1 elements.

### Statistical analysis

To account for correlated outcomes among boilermakers who participated in multiple sampling cycles, we used linear mixed-effects regression models with random intercepts to investigate 1) the effect of PM_2.5_ on HRV, 2) the effect of PM_2.5_ on DNA methylation and 3) whether DNA methylation served as a mediator of the PM_2.5_ effect on HRV.

#### Effect of PM _2.5_ on HRV

For each participant, we measured pre- and post-resting HRV outcomes over five minutes for each sampling cycle. Recordings were analyzed in both the time and frequency domains, and SDNN, rMSSD, pNN10, pNN20, LF, and HF were calculated. We used a linear mixed-effect model with random intercepts to account for correlated outcome measures repeatedly collected from the same subject. We estimated the acute effect of PM_2.5_ on HRV outcomes using the following model:
1

where for the fixed effects, β_0_ is the overall intercept; β_1_ is the regression coefficient representing the effect of PM_2.5_ on HRV outcomes; β_2_ … β_p_ are the regression coefficients for the covariates included in adjusted models; i = 1, 2,…, 66 represents the subject; j = 1, 2,…6 represents the j^th^ sampling cycle; k = 1, 2 in the outcome variable represents pre- or post-welding samples, while the measurement of PM_2.5_ represents background or welding PM exposures. The random intercept for each subject is b_i_ and the residual error term is e_ijk_. Adjusted models included the covariates age, starting time of the day (am/pm), season, sampling year, current smoking status (yes/no), and exposure to second-hand smoke (yes/no). Restricted maximum likelihood method was used for estimation. Significance of fixed effects was based on the Wald tests [31].

#### Effect of PM _2.5_ on DNA methylation

For each participant, we measured DNA methylation in two blood samples (pre- and post-exposure) collected in each sampling cycle. For each blood sample, the average DNA methylation levels of both Alu and LINE-1 elements were calculated over five and four CpG dinucleotide positions, respectively. The effect of PM_2.5_ on DNA methylation levels measured as %5mC was estimated using the same model (1). Adjusted models included the following covariates as fixed effects: age, starting time of day (am/pm), season, batch effect of methylation analysis, current smoking status (yes/no), and exposure to second-hand smoke (yes/no).

#### Conditional effect of LINE-1 methylation on HRV

The effect LINE-1 methylation levels on HRV after controlling for PM_2.5_ was estimated with model (2):
2

where for the fixed effects, β_0_ is the overall intercept; β_1_ is the regression coefficient representing the effect of LINE-1 methylation on HRV outcomes after adjusted for PM_2.5_, the conditional effect of PM_2.5_ was estimated as β_2_; and β_3_ … β_p_ are the regression coefficients for the covariates included in adjusted models. We included the same covariates as in the analysis of the PM_2.5_ effect on HRV.

## Results

### Personal exposure levels

Average personal exposure levels of PM_2.5_ are shown in Table 2. The average welding shift was five hours. PM_2.5_ levels from the background environment were an average of 0.11 mg/m^3^ (SD = 0.14 mg/m^3^), while PM_2.5_ during welding were an average of 0.43 mg/m^3^ (SD = 0.34 mg/m^3^). These differences are statistically significant (p < 0.0001).

**Table 2:** **Summary of PM**_**2.5**_ **, HRV, and DNA methylation in boilermakers**

	Mean (SD)		Difference in Measurement	p-value ^a^
	Background Environment	Welding exposure	Welding – Background	
Personal PM_2.5_ (mg/m^3^)	0.11 (0.14)	0.43 (0.34)	0.32	<0.0001
			Difference in Measurement	
	Pre-shift	Post-shift	Post-shift – Pre-shift	p-value^a^
Alu (%5mC)	29.4 (1.1)	29.5 (1.06)	0.10	0.396
LINE-1 (%5mC)	82.5 (1.7)	82.7 (1.7)	0.22	0.240
SDNN (msec)	48.2 (24.2)	37.8 (18.7)	-8.7	<0.0001
rMSSD (msec)	26.5 (19.1)	21.1 (11.9)	-4.45	0.001
pNN10 (%)	51.4 (22.0)	42.5 (22.1)	-0.08	0.0002
pNN20 (%)	33.2 (22.6)	25.8 (20.8)	-0.07	0.002
LF (msec^2^)	1019.4 (1289.0)	593.0 (561.9)	-378.3	0.0003
HF (msec^2^)	454.6 (884.3)	194.6 (284.3)	-228.9	0.001

^a^ Accounting for within subject correlation.

### A subgroup population and baseline HRV variation

A subgroup of 47 male boilermakers were monitored for the resting HRV on non-welding days prior to any welding tasks. This subgroup of male boilermakers have similar mean age, BMI and race distribution as the main welding group (Table 3). However, the current smoking rate is 61.2% whereas 43.1% of smokers in the welding group.

**Table 3:** **Summary of a subgroup population characteristics**

Characteristics (n = 49)		Mean (SD)
Age (years)		42 (11.6)
Range		22-71
BMI (kg/m^2^)		28.0 (5.1)
Range		16.9-38.7
Race	N	%
White	39	80
Black	6	12
Hispanic	2	4
Asian	2	4
Male	49	100
Current smoker	30	61.2

Average HRV measurements are shown in Table 4. The differences of HRV measurements between pre and post-shift are not statistically significant (p > 0.05). Whereas in Table 2, the decline of HRV measurements after welding exposure are statistically significant (p < 0.05).

**Table 4:** HRV measurements of a subgroup on non-welding days (n = 49)

	Mean (SD)		Difference in Measurement	p-value ^a^
	Pre-shift	Post-shift	Post-shift – Pre-shift	
SDNN (msec)	45.4 (23.5)	45.5 (23.1)	1.9	0.32
rMSSD (msec)	26.5 (18.5)	26.4 (189.3)	0.69	0.65
pNN10 (%)	51.97 (22.0)	50.1 (22.9)	-0.56	0.75
pNN20 (%)	34.1 (23.2)	32.1 (23.8)	-0.65	0.7
LF (msec^2^)	850.3 (976.1)	853.4 (888.2)	60	0.43
HF (msec^2^)	427.6 (804.5)	407.4 (719.2)	3.23	0.97

^a^Accounting for within subject correlation.

### Effect of PM_2.5_ on HRV

In unadjusted models, PM_2.5_ was associated with decreased HRV measured in natural log-transformed SDNN, rMSSD, LF, HF and natural percentage pNN10 and pNN20 (Table 5). In covariate-adjusted models, PM_2.5_ was still associated with decreased pNN10, pNN20 and ln HF (β = -0.16%, p = 0.006; β = -0.13%, p = 0.030 and β = -0.76, p = 0.035, respectively). The effect of PM_2.5_ on heart rate variability was interpreted as follows: with each 1 mg/m^3^ increase in PM_2.5_ exposure level, there was a 17% decrease in pNN10, 13% decrease in pNN20, and 55% decrease in power in the high-frequency range, adjusted for age, starting time of the day (am/pm), season, current smoking status (yes/no), and exposure to second-hand smoke.

**Table 5:** **Effect of PM**_**2.5**_ **on HRV**

	Unadjusted regression			Adjusted regression ^a^		
	β	95% CI	p-value	β	95% CI	p-value
SDNN ^b^ (msec)	-0.31	(-0.54 ~ -0.08)	0.008	-0.20	(-0.46 ~ 0.07)	0.138
rMSSD ^b^ (msec)	-0.28	(-0.52 ~ -0.04)	0.024	-0.20	(-0.49 ~ 0.08)	0.157
pNN10 (%)	-0.16	(-0.26 ~ -0.06)	0.002	-0.16	(-0.28 ~ -0.05)	0.006
pNN20 (%)	-0.14	(-0.24 ~ -0.04)	0.008	-0.13	(-0.25 ~ -0.01)	0.030
LF ^b^ (msec^2^)	-0.59	(-1.07 ~ -0.11)	0.017	-0.43	(-0.98 ~ 0.13)	0.127
HF ^b^ (msec^2^)	-0.80	(-1.40 ~ -0.20)	0.008	-0.76	(-1.46 ~ -0.05)	0.035

^a^ Adjusted for age, starting time of the day, season, sampling year, current smoking status, and second-hand smoke exposure.

^b^ The outcome measurements were natural log (ln)-transformed.

### Effect of PM_2.5_ on DNA methylation

We found that PM_2.5_ had a crude positive association with LINE-1 methylation levels [β = 0.81 (%5mC), p =0.024]. The association was significant after adjustment for covariates of age, starting time of the day (am/pm), season, current smoking status (yes/no), and second-hand smoke exposure [β = 0.79 (%5mC), p = 0.013]. The association of PM_2.5_ and Alu methylation levels was also positive but not statistically significant from both crude and adjusted analysis [β = 0.21 (%5mC), p = 0.376 and β = 0.06 (%5mC), p = 0.767]. Results are shown in Table 6.

**Table 6:** **Effect of PM**_**2.5**_ **on DNA methylation**

Transposable elements	Unadjusted regression			Adjusted regression ^a^		
	β	95% CI	p-value	β	95% CI	p-value
Alu (%5mC)	0.21	(-0.26 ~ 0.67)	0.376	0.06	(-0.34 ~ 0.46)	0.767
LINE-1 (%5mC)	0.81	(0.11 ~ 1.51)	0.024	0.79	(0.18 ~ 1.42)	0.013

^a^ Adjusted for age, starting time of the day, season, batch effect, current smoking status, and second-hand smoke exposure.

### Conditional effect of LINE-1 methylation on HRV, adjusting for PM_2.5_

To further test the mediation pathway of PM-induced LINE-1 methylation changes on HRV, we investigated the effect of LINE-1 methylation levels on HRV measurements controlled for PM_2.5_ exposure. In the crude analysis adjusting only for PM_2.5_, the results showed a positive association between LINE-1 and ln rMSSD, ln LF, and ln HF (β = 0.05, p = 0.020; β = 0.09, p = 0.026 and β = 0.10, p = 0.043, respectively). However, after adjusting for PM_2.5_ and covariates of age, starting time of the day, season, sampling year, current smoking status, and second-hand smoke, the association between LINE-1 methylation and HRV was not statistically significant (Table 7).

**Table 7:** **Conditional effect of LINE-1 methylation on HRV, controlled for PM**_**2.5**_

	Unadjusted regression			Adjusted regression ^a^		
	β	95% CI	p-value	β	95% CI	p-value
SDNN ^b^ (msec)	0.04	(-0.000 ~ 0.08)	0.053	0.02	(-0.04 ~ 0.07)	0.514
rMSSD ^b^ (msec)	0.05	(0.01 ~ 0.08)	0.020	0.04	(-0.01 ~ 0.10)	0.130
pNN10 (%)	0.01	(-0.002 ~ 0.03)	0.085	0.02	(-0.01 ~ 0.04)	0.189
pNN20 (%)	0.01	(-0.004 ~ 0.03)	0.128	0.01	(-0.01 ~ 0.03)	0.442
LF ^b^ (msec^2^)	0.09	(0.01 ~ 0.17)	0.026	0.05	(-0.06 ~ 0.16)	0.336
HF ^b^ (msec^2^)	0.10	(0.004 ~ 0.20)	0.043	0.10	(-0.04 ~ 0.24)	0.162

^a^ Adjusted for age, starting time of the day, season, sampling year, current smoking status, and second-hand smoke exposure.

^b^ The outcome measurements were natural log (ln)-transformed.

## Discussion

We demonstrated a statistically significant effect of PM_2.5_ on a reduction in HRV measured by HF, pNN10, and pNN20, and in SDNN and rMSSD that showed a marginally significant effect. Our results confirm an inverse exposure-response relationship between PM_2.5_ exposure and HRV and further support evidence of an observed short-term HRV change following an average of five hours of welding exposure.

These findings also confirm a previous study showing a steep short-term decline in hourly SDNN index (SDNNi) in the first few hours post-exposure to welding PM_2.5_, followed by a plateau and a second period of decline in the 9–10 hours post-exposure [28]. The variability of results seen in studies where PM_2.5_ induces HRV alterations may be due to differences in sources and components of PM_2.5_. A study of highway patrol troopers with exposure to PM_2.5_ originating from traffic combustion showed a post-shift increase in SDNN and pNN50 [32]. The assessment of PM_2.5_ metal exposure from another study confirmed the inverse relationship between manganese (Mn) and night-time rMSSD [33]. However, with increases in lead and vanadium (Pb and V) concentration, statistically significant mean increases in day-time SDNNi were also reported from the same cohort [13].

A subset of the participants (47 out of 66 participants) were also monitored on non-welding days prior to welding days. We tested the differences between pre and post-shift HRV measurements and the results did not show significant changes between pre and post-shift HRV (Table 4.). However, the higher prevalence of current smokers in this subgroup may be a confounding factor in this non-significant differences between pre and post-shift HRV on non-welding days. Due to incomplete data, we were not able to control the baseline HRV measurements for all participants in the final model. Based on the results and the assumption of missing at random, we assumed the variability of observed HRV measurements on welding days were unlikely due to circadian variation which is not confounding the observed negative effect of PM_2.5_ in this study.

Transition metal components of PM_2.5_ are inhaled and delivered into the airways and can catalyze the Fenton reaction to generate reactive oxygen species (ROS), leading to oxidative stress, although the exact mechanism remains unclear [34]. Oxidative stress can cause endothelial injury and inflammation followed by cardiac autonomic dysfunction, which can then be visualized in an altered heart rate pattern [35]. Recent studies have suggested that oxidative stress as a consequence of ROS accumulation induces epigenetic profile alterations in peripheral blood leukocytes to further interfere with DNA, leading to changes in gene expression and, eventually, adverse cardiovascular outcomes [19, 21]. In addition, welding particles has been associated with increased systemic inflammation and study has reported that DNA methylation is associated with ROS and inflammatory exposure [7, 36]. In this study we investigated the epigenetic effect of welding PM_2.5_, and found a significant association between welding PM_2.5_ and increased blood methylation level of LINE-1. Transposable element LINE-1 has more complete retrotransposon structures thus it has different biological functions from Alu, and studies have suggested that Alu and LINE-1 methylation responded differently to environmental factors [17, 37]. Our results suggest that LINE-1 might be more sensitive to short-term exposure whereas Alu is more susceptible to cumulative exposure over time [7]. We tested the association between blood cell types and blood methylation levels in Alu and LINE-1 and found that the percentage of either neutrophils or lymphocytes, which are the major differentials in peripheral blood, was not significantly associated with methylation levels. Hence, although DNA methylation is cell-type specific, our analysis shows that blood cell differentials were not confounding the observed epigenetic effect of PM_2.5_ in this study.

It is generally understood that PM-induced oxidative DNA damage can interfere with the ability of DNA methyltransferase to interact with DNA to reduce methylation [38]. However, one study showed an increase in global methylation in sperm from mice exposed to particulate air pollution in an urban/industrial location, supporting the positive association of global hypermethylation and particulate exposures [39]. Along with our results, the positive association between LINE-1 methylation and welding PM_2.5_ suggests other factors that may play a role in PM-induced ROS. Another possible explanation may be the complex toxicity of welding exposure compositions. For example, a coke-oven worker study has reported a significant association between increased methylation of LINE-1 and exposure to PAHs [40], which are also generated from welding processes and whose genotoxic risk is well established [41]. In addition to organic chemicals, nickel-induced higher global methylation was also found in Chinese hamster G12 cells [42]. Unfortunately, there is a limitation in this study that concomitant pollutants including manganese, nickel and chromium from the practicing on a mix of standard and stainless steel welding are potential confounders that we were unable to control the unmeasured pollutants or perform a compositional exposure assessment of to further extricate the observed epigenetic responses to welding fume. In addition, smoking was only controlled as a dichotomous variable because the smoking behavior information was collected through a lifestyle questionnaire so that the lack of accuracy might not provide us to evaluate the effect of smoking measured in quantity. Also, the possible influences of electromagnetic fields generated from welding activities (in the absence of welding fume exposures) on the HRV measurements has not been assessed.

Many studies have shown a link between altered repetitive element methylation and cardiovascular diseases [23], yet the underlying epigenetic regulatory pathways have not been identified. To understand the role of DNA methylation between PM exposure and reduced HRV, we performed mediation analysis [43] to investigate whether LINE-1 methylation is a mediator of the association between PM_2.5_ exposure and HRV outcomes. This method allowed us to decompose a total effect of exposure on an outcome into a direct effect of the exposure and an indirect effect of the exposure through a mediator’s pathway [44]. Mediation analysis usually requires a significant association between the exposure and the mediator, and a significant association between the mediator and the outcome [43]. However, the data presented here show a non-significant positive association between LINE-1 methylation level and HRV after adjustment of PM_2.5_, therefore we did not see any significant mediation effect. The association between LINE-1 methylation and HRV was tested after a few hours of welding exposure, which might not capture the best timing of a dose–response relationship but still suggests a link between decreased LINE-1 methylation and adverse cardiac outcomes. A limitation of this study is the impossibility of collecting tissue-specific heart cells from human subjects and lack of data on gene-specific methylation such as genes involved in regulating oxidative stress and inflammatory, since LINE-1 is a transposable element and lacks the specificity necessary to serve as a mediator in a biological pathway. In addition, the relatively small sample size of this study, though adequate for repeated-measures panel results, may be a limitation in estimating the mediation effect.

## Conclusions

In summary, our results show the acute decline of HRV following the exposure of metal-rich welding PM_2.5_ and support evidence of a short-term cardiac response to welding exposure. We also show a short-term increase in DNA methylation in LINE-1 elements following welding exposure. These results support a systemic epigenetic response to short-term exposure to high-level metal-rich particulates, however no mediation effect of LINE-1 methylation was supported from the study. Further studies involving specific tissues and gene methylation are required to establish a more detailed mechanism for the observed cardiac responses.

## Electronic supplementary material

Additional file 1: Table S1: Primers and PCR conditions for DNA methylation analysis. (DOC 33 KB)

## Abbreviations

PM_2.5_: Fine particulate matter
HRV: Heart rate variability
LINE-1: Long interspersed nuclear element-1
SDNN: The standard deviation of the NN intervals
rMSSD: Square root of the mean squared difference of successive NN intervals
pNN10: The proportion of the number of interval differences of successive NN intervals greater than 10 milliseconds over the total number of NN intervals
pNN20: The proportion of the number of interval differences of successive NN intervals greater than 20 milliseconds over the total number of NN intervals
LF: Power spectral density in the low frequency (0.04–0.15 Hz)
HF: Power spectral density in the high frequency (0.15–0.40 Hz)
ROS: Reactive oxygen species.
