# Supplementary material for: Heart rate variability and DNA methylation levels are altered after short-term metal fume exposure among occupational welders: a repeated-measures panel study
Source: BMC Public Health. 2014 Dec 16;14:1279. doi: 10.1186/1471-2458-14-1279 (PMC4302115; doi:10.1186/1471-2458-14-1279)
Supplement: Supplementary file 1 — Additional file 1: Table S1: Primers and PCR conditions for DNA methylation analysis. (DOC 33 KB) [file 12889_2014_7387_MOESM1_ESM.doc]

Additional file 1

Title: Cardiovascular function and DNA methylation levels are altered after short-term metal fume exposure among occupational welders

Authors list:

Tianteng Fan ^1^, Shona C.Fang^1,3^, Jennifer M. Cavallari^1,4^, Ian J. Barnett^2^, Zhaoxi Wang^1^, Li Su^1^, Hyang-Min Byun,^1^ Xihong Lin^2^, Andrea A. Baccarelli^1,5^, David C. Christiani^1,5,6^

^1^ Department of Environmental Health, Harvard School of Public Health, Boston, Massachusetts, USA

^2^ Department of Biostatistics, Harvard School of Public Health, Boston, Massachusetts, USA

^3^ New England Research Institute, Watertown, Massachusetts, USA

^4^ University of Connecticut Health Center, Farmington, Connecticut, USA

^5^ Department of Epidemiology, Harvard School of Public Health, Boston, Massachusetts, USA

^6^ Pulmonary and Critical Care Unit, Massachusetts General Hospital/Harvard Medical School, Boston, Massachusetts, USA

Address correspondence to David C. Christiani, Department of Environmental Health, Harvard School of Public Health, Boston, Massachusetts, USA 02115. Telephone: 6174323323. Email: [dchris@hsph.harvard.edu](mailto:dchris@hsph.harvard.edu).

Table S1. Primers and PCR conditions for DNA methylation analysis

| Sequences | Forward primer (5' to 3') | Reverse primer (5' to 3') | Sequencing primer (5' to 3') | PCR conditions |
| --- | --- | --- | --- | --- |
| Alu | Biotin-TTTTTATTAAAAATATAAAAATT | CCCAAACTAAAATACAATAA | AATAACTAAAATTACAAAC | 96°C for 90 s, 43°C for 60 s, 72°C for 120 s (40 cycles) |
| LINE-1 | TTTTGAGTTAGGTATGGATATA | Biotin-AAAATCAAAAAATTCCCTTTC | AGTTAGGTGTGGGATATAGT | 95°C for 30 s, 50°C for 30 s, 72°C for 30 s (35 cycles) |

References

1. Bollati V, Baccarelli A, Hou L, Bonzini M, Fustinoni S, Cavallo D, Byun HM, Jiang J, Marinelli B, Pesatori AC, Bertazzi PA, Yang AS: **Changes in DNA methylation patterns in subjects exposed to low-dose benzene.** *Cancer Res* 2007.**67:** 876-880.
